# Young Adults with a History of Residential Youth Care: A Cohort Profile of a Hard-to-Reach Population

**DOI:** 10.3390/ijerph21111447

**Published:** 2024-10-30

**Authors:** Hanne Klæboe Greger, Maria C. Stuifbergen, Thomas Jozefiak, Nanna Sønnichsen Kayed, Stian Lydersen, Tormod Rimehaug, Inga Schalinski, Astrid Røsland Seim, Marianne Tevik Singstad, Jan Wallander, Lars Wichstrøm, Stine Lehmann

**Affiliations:** 1 Department of Mental Health, Norwegian University of Science and Technology, 7491 Trondheim, Norway; maria.stuifbergen@ntnu.no (M.C.S.); thomas.jozefiak@ntnu.no (T.J.); nanna.kayed@ntnu.no (N.S.K.); stian.lydersen@ntnu.no (S.L.); tormod.rimehaug@ntnu.no (T.R.); astrid.r.seim@ntnu.no (A.R.S.); marianne.t.singstad@ntnu.no (M.T.S.); jwallander@ucmerced.edu (J.W.); 2 Department of Mental Healthcare—Emergency and Children, St. Olavs Hospital, 7006 Trondheim, Norway; 3 Department of Human Sciences, Universität der Bundeswehr München, 85579 Neubiberg, Germany; inga.schalinski@unibw.de; 4 Department of Psychological Sciences, University of California, Merced, CA 95343, USA; 5 Department of Psychology, Norwegian University of Science and Technology, 7491 Trondheim, Norway; lars.wichstrom@ntnu.no; 6 Department of Clinical Psychology, Faculty of Psychology, University of Bergen, 5020 Bergen, Norway; stine.lehmann@uib.no

**Keywords:** residential youth care, child welfare institution, emerging adults, recruitment, hard-to-reach population, out-of-home care

## Abstract

Adults with a history of living in residential youth care (RYC) face elevated risks across various life domains. In this cohort profile paper, we outline the design of a comprehensive follow-up study—the VINGO study—targeting young adults (22–30 years) with a history of living in RYC (T2). We describe the recruitment strategy and present sample characteristics. Data were collected in the baseline study (T1) from 2011 to 2014. At T1, the 400 adolescent participants showed a high prevalence of mental disorders, maltreatment experiences, substance use, and self-reported suicide attempts. Data collection at T2 10 years later (2021–2023) included self-reported sociodemographic information, physical health, childhood maltreatment, dissociation, quality of life, social support, and self-esteem using standardized and validated instruments. A diagnostic psychiatric assessment and subjective evaluation of service utilization were conducted by telephone interviews. Additionally, a qualitative sub-study involved in-depth interviews of fourteen participants. We reached a 52% response rate at T2. Comparing participants (n = 157, 107 females) to non-participants (n = 243, 123 females) based on T1 data revealed that T2 participants had a higher prevalence of depression, anxiety, and conduct disorder and a lower prevalence of ADHD at T1. Furthermore, T2 participants reported more suicide attempts, experiences of maltreatment, and problematic substance use at T1. Our results show that we reached a burdened population, positioning the VINGO study as a unique opportunity to examine a vulnerable population of emerging adults.

## 1. Introduction

In this cohort profile paper, we aim to present the protocol of the VINGO study (Adults with Residential Youth Care History—Good Transitions) and the results of the recruitment strategy, including sample characteristics. In 2011–2014, we conducted a nationwide study of mental health in adolescents in residential youth care (RYC) in Norway [1]. The current study is a ten-year follow-up of the participants from the first wave. The main objective of the VINGO study is to investigate the functioning of young adults who have lived in RYC during adolescence, focusing on physical and mental health, psychosocial functioning, quality of life (QoL), and the use of and experiences with a wide range of health and social services. Previous studies have found that youths placed in RYC are at risk of various disadvantageous outcomes, including a high rate of mental disorders [2], a high risk of poor educational outcomes [3,4], and poor QoL [5]. Upon entering young adulthood, a substantial proportion struggles to enter work life [6]. Moreover, they are at continued risk for adult mental health problems [7,8,9,10], physical health problems [11,12], substance abuse [13,14], premature death [15,16], incarceration [17,18], and early disability benefits [6]. However, less is known about their access to relevant services, whether the services are adapted to their special needs, and whether service use and experiences can mediate or moderate improvements in health and adult functioning. Therefore, the VINGO study can connect their history of health and functioning in adolescence and emerging adulthood with their use and experience of health and social services.

There is a mismatch between the human and economic resources devoted to RYC on the one hand and the lack of knowledge of long-term outcomes and the need for services on the other. In the general population, longitudinal studies have shown that mental disorders in childhood or adolescence increase the risk of both homotypic and heterotypic continuity of mental disorders in early adulthood [19]. However, there is little knowledge about the continuity across health and psychosocial dimensions among youths in RYC institutions. This is important information to plan best practices in health and social services to prevent the development of poor health and QoL. This, in turn, can lead to positive economic, educational, and social outcomes for this population. This knowledge gap is maintained by the repeated experience that this vulnerable group is hard to reach and recruit for research purposes. The VINGO study was planned to provide broad knowledge about the health, QoL, psychosocial functioning, and service needs and experiences of a hard-to-reach population. It provides a unique opportunity to increase current knowledge of the transition to adulthood of young people in RYC.

### 1.1. Residential Youth Care in the Norwegian Child Welfare Context

The child welfare system in Norway is mainly service-oriented, emphasizing preventive in-home measures to enhance parental abilities to support their children’s development. In addition to the family-centered approach, the Norwegian child welfare system is also child-centered, where the child is seen as an individual with interests and opinions that they should have the opportunity to express [20]. Based on public registries, there are approximately 1000 children and adolescents in RYC institutions in Norway at any timepoint, although this number has been decreasing over time. The majority of youth in RYC have entered out-of-home care due to abusive and/or neglectful family environments [21]. In Norway, placement in foster families is considered the first choice when out-of-home care is needed. Still, RYC remains an option, especially for adolescents with conduct problems or problems with substance use.

RYC institutions approved by the Norwegian Directorate of Children, Youth and Family Affairs can be commercial, non-profit, or public enterprises. These institutions are typically specialized in different areas: acute placements, care, conduct problems, or substance use problems. Some are in rural areas, while others are in small towns or larger cities. Most Norwegian RYC institutions are small units with three to five residents, where the adolescents are encouraged to sustain normal activities, attend school, and participate in leisure activities [1]. Placements in RYC can be with or without parental or youth consent based on decisions by the Child Welfare Tribunal according to the Child Welfare Act [22]. Results from our baseline study showed that several of the adolescents in RYC had experienced multiple placements by child welfare services (CWSs), with one out of three reporting more than three placements at the time of the study [23].

### 1.2. Characteristics of Young People in Residential Youth Care and Summary of Results from the Baseline Study

The baseline study (T1) was conducted in 2011–2014 when the participants were 12 to 20 years old (n = 400). Overall, our results revealed that 76% of the adolescents met the criteria for at least one diagnosis according to the Diagnostic and Statistical Manual of Mental Disorders, Fourth Edition (DSM-IV), during the last three months. The most prevalent disorder category was depressive disorders (37%), followed by anxiety disorders (34%) and Attention Deficit Hyperactivity Disorder (ADHD) (32%). A high rate of comorbidity was observed [1,24]. Two types of trauma- and stressor-related disorders, which have a low prevalence in both the general population and psychiatric services, were not uncommon, with a prevalence of 9% for Reactive Attachment Disorder and 8% for Disinhibited Social Engagement Disorder [25]. Problematic substance use was reported by 56% of the adolescents [24]. Only 38% had accessed mental health services during the last 3 months [23]. Moreover, adolescents in RYC reported poorer QoL [5,26] and less social support [27] compared to adolescents in the general population. However, they generally expressed satisfaction with the social support they received [27]. This is important because perceived social support is associated with lower symptom loads of mental health problems [28] and higher QoL [29] in this group. A total of 71% of the adolescents reported exposure to at least one type of maltreatment. These experiences were associated with an increased risk of mental disorders, previous suicide attempts [30], and poor QoL [31].

### 1.3. Emerging Adulthood

The recruitment period for our follow-up study coincided with the phase of emerging adulthood for the participants. The term emerging adulthood defines the developmental period following adolescence and prior to “full” adulthood, generally spanning the ages from 18 to 29 years [32]. It is characterized by changes in several life areas, often with instability in romantic relationships, work, and habitation, and represents the period where most individuals find their way to full independence from caregivers [33]. Research has shown that brain development continues well into the third decade of life, indicating that emerging adults may have immature abilities of executive functions, cognition, and the integration of emotions [34]. During this developmental period, most individuals have not yet fully achieved goals and obligations associated with adulthood, such as stable work, financial stability, stable long-term intimate relationships, and family life and parenting. The challenges and potentials of emerging adulthood are closely linked to the culture and economy of the society [33], leading to different expectations and possibilities. Especially in Western countries, emerging adulthood is the period where individuals leave the family home, seek to find their way through education and/or work life, establish a more long-term romantic relationship, and make important life choices. Emerging adulthood is characterized by less adult supervision and monitoring. For some, this could open up possibilities for risky behavior including binge drinking, high-speed driving, illicit drug use, and unprotected sexual activity [33,35].

The normal instability of emerging adults may cause challenges to public services as they are no longer minors according to law yet not yet fully independent. They might need assistance in making important decisions related to their own health and life situation [32]. Due to the high prevalence of mental health problems and lower level of social support from their families, adolescents with a history of living in RYC institutions can therefore be expected to face a more challenging transition to adulthood compared to adults raised without experience of out-of-home care. Given the high prevalence of instability in their life situation in this period, young adults with prior RYC experience are often difficult to connect with and recruit for a follow-up study. Studies on adolescents leaving residential youth care are predominantly conducted in the US, with limited studies in European settings [36].

Adolescents in RYC are facing transitions not only from dependency to more independent living arrangements but also from compulsory school to work or advanced education. Many of them will also need to interact with healthcare and social services to manage their ongoing needs [37,38], all within a frequently shifting and temporary care setting. These young adults’ capacity and skills to navigate and interact with public services may be impaired by mental health problems, which can affect their long-term outcomes. Accordingly, CWSs in Norway are obliged to offer aftercare to individuals in RYC during the ages of 18–25 years in the transition to independent adulthood [22]. The content and duration of aftercare are based on consent and can be individually tailored, with the aim being to ease the transition to adulthood by targeting the needs of the individual. Aftercare can involve several kinds of support and services, including counseling, prolonged care, financial support, or the establishment of other necessary services. Participation in aftercare in the form of transitional programs focusing on housing, education, and employment may improve life outcomes [39]. However, there are no standard procedures for the delivery of aftercare in Norway, and, therefore, such services vary across regions and municipalities [40].

### 1.4. Objectives

The overall aims of the VINGO study are to increase knowledge of individual and contextual factors that contribute to successful transitions during the emerging adulthood period among youths in RYC institutions. We investigate their subjective experiences and current health and life situations as adults. We hypothesize that a substantial number of emerging adults transitioning from RYC will encounter extensive challenges related to mental and physical health, education and employment, personal economy, social relations, and QoL. We therefore also hypothesize that this group will need interventions from multiple services. This will be examined in a series of sub-studies. A list of the main topics, study aims, and examples of planned research questions are listed in Table 1.

The present cohort paper has three objectives:

1. To outline the research protocol for the VINGO follow-up study.
2. To present the details of the recruitment procedures and outcomes of these efforts with this hard-to-reach population with a high risk of aberrant outcomes.
3. To examine the representativeness of the sample of the VINGO follow-up study through attrition analysis.

## 2. Materials and Methods

### 2.1. Design and Procedure

#### 2.1.1. The Baseline Study (T1)

**Figure 1:**
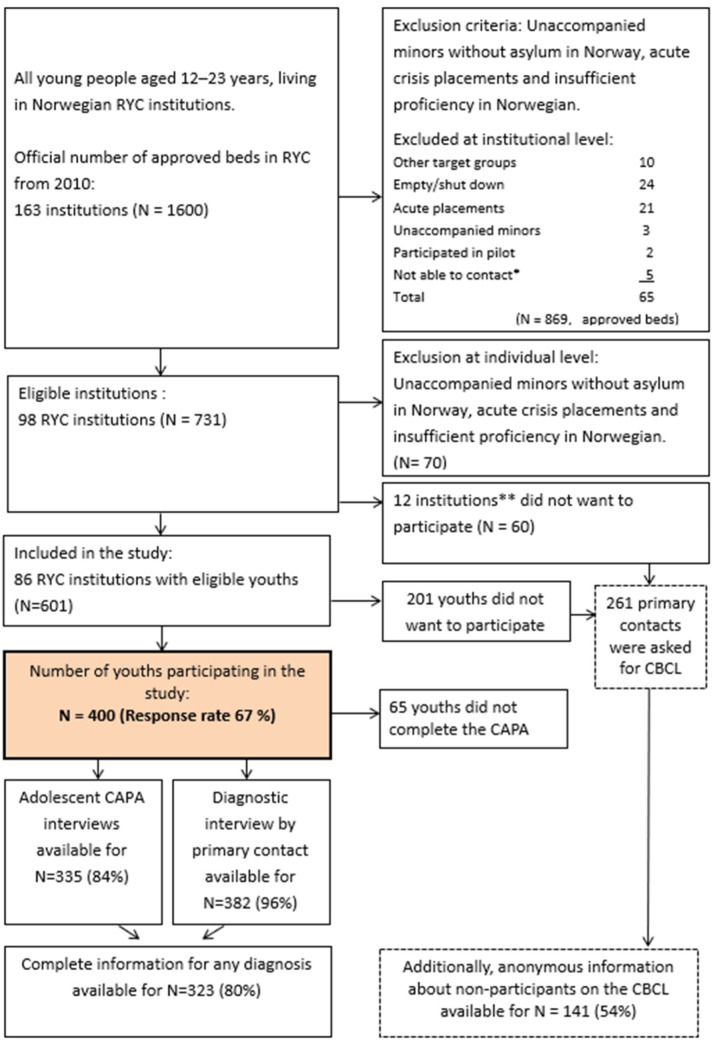
Flowchart of baseline recruitment. * The category “not able to contact” was used if institutional staff did not respond to repeated approaches about participation over a period of several months. ** There were no significant differences between participating and non-participating RYC institutions with regard to geography and ownership.

During the period of June 2011–July 2014, adolescents in RYC institutions in Norway were invited to participate, with some exclusion criteria (see Figure 1) [1].

A total of 400 out of 601 eligible adolescents participated (67% response rate, mean age girls = 16.5 years, mean age boys = 16.9 years). The baseline study included a semi-structured diagnostic interview assessing mental health, an interview about their child welfare background, a neuropsychological test of executive functions, and a questionnaire concerning their perception of RYC, psychological functioning, quality of life, and social relations (for a detailed description, see [1]). All 400 participants from baseline consented to be contacted for follow-up research. Personal identification numbers used in official Norwegian registrations (PINs) were retained from the participants.

#### 2.1.2. The VINGO Follow-Up Study (T2)

At T2, 10 years post T1, PINs were coupled with an official registry containing phone numbers and e-mail addresses. Home addresses were obtained from the National Population Registry. Contact with eligible participants was initiated by an SMS containing brief information about the study and a link to an online information and consent form. The young adult was invited to participate by completing an online survey and a subsequent telephone interview. Within a week, the study coordinator made a phone call to explain the purpose of the study and invite potential participants to partake in the study. Attempts to make contact through SMS and phone calls were repeated until contact was obtained or three unsuccessful attempts had been made. If no response was obtained, an information letter with an invitation to participate was sent via both e-mail and postal mail. For those who completed the online survey, the coordinator made contact to make an appointment for the telephone interview. The recruitment period for the VINGO study was 5 January 2021–17 April 2023. Participants received a gift card (NOK 500 = EUR 44) after they completed the telephone interview. All participants were also included in a lottery for a larger value gift card (NOK 10,000 = EUR 875).

### 2.2. Assessments and Measures

#### 2.2.1. Quantitative Sub-Study

The quantitative sub-study comprised an online survey and a structured interview conducted over the telephone.

##### Survey

The main domains addressed in the survey were sociodemographic information, education, employment, family relations, physical health and COVID impact, childhood maltreatment, dissociation, quality of life, social support, self-esteem, psychosocial functioning, and service use and experiences. A detailed overview of domains, measures, and items is provided in Table 2.

##### Interview

A semi-structured psychiatric assessment using the M.I.N.I. international neuropsychiatric interview (M.I.N.I plus) [42] was conducted during a telephone interview. To secure an optimal diagnostic evaluation, interviewers were recruited among clinical psychologists and medical doctors with experience of mental health services and diagnostic competence. A total of nine interviewers participated in digital meetings for discussion and training. The telephone interviews were recorded for quality purposes and to analyze interrater reliability.

The M.I.N.I. plus sections included in the current study were depression, dysthymia, suicidality, mania, panic disorder, agoraphobia, social phobia, specific phobia, obsessive-compulsive disorder, alcohol and drug use/dependency, psychosis, eating disorder, generalized anxiety disorder, somatoform disorder, somatoform pain disorder, and ADHD. The interviewers made diagnostic conclusions exclusively based on the participants’ answers to the M.I.N.I plus interview. In addition, post-traumatic stress disorder (PTSD) was assessed using the Posttraumatic Stress Disorder Checklist for DSM-5 (Norwegian version; TRAPS) [44,45], personality traits were assessed with 102 items from the DSM and ICD Personality Questionnaire [49,50,51], and subjective experience of service use was assessed with adapted questions from the Child and Adolescent Services Assessment (CASA) interview [67,68]. A timeline to address the participants’ housing arrangements since living in RYC was also established. Quality control of the data files revealed missing data from two of the recorded telephone interviews. In these cases, one of the interviewers re-scored information from recordings to complete the data files.

#### 2.2.2. Qualitative Sub-Study

To provide supplementary information regarding participants’ experiences with services and inter-service cooperation, semi-structured, in-depth, qualitative interviews were conducted with 14 young adults. To ensure variation in participant characteristics, selection criteria for participation were established, including sex, age, region of residence, and diverse satisfaction with services, as assessed in the survey. Based on these criteria, the research team created a list of 18 potential participants and initiated contact with them. Among the young adults, 14 consented to participate in the qualitative interview. Respondents were given the choice of four alternatives for the interview: (1) telephone, (2) digital platform (with or without camera), (3) physical interview near the respondent’s place of residence, or (4) physical interview at the research institution in Trondheim. Each interview had a maximum duration of 90 min. A semi-structured interview guide was developed, with overall categories concerning experiences with services (both positive and negative), inter-service cooperation, and practical implications for optimal services for young adults with a history of living in RYC. All qualitative interviews were conducted between 3 March 2023 and 10 May 2023. As compensation for their participation, the respondents received an additional gift card (NOK 500 = EUR 44).

#### 2.2.3. Register-Based Data

To complement self-reported data and include objective measures, this study gained permission from the Norwegian Agency for Shared Services in Education and Research to merge data from Norwegian population registries. This includes data from the Medical Birth Registry of Norway, the Norwegian Patient Registry, the Norwegian Prescription Database, the Sanction Register, and the National Insurance Administration Registry. Relevant registry data will be included in subsequent papers disseminating results from the VINGO study.

### 2.3. Analyses

All data, written and recorded, were directly transferred to and stored on a secure server (services for sensitive data (TSD)) administered by the University of Oslo. In the current paper, we present sample characteristics by means (standard deviations) and percentages. Analyses were conducted in SPSS version 29.0.1.0 (IBM Corp, Armonk, NY, USA) (171).

### 2.4. Ethical Consideration

The baseline study was approved by the regional ethical committee (REK) (2010/1965/REK Midt) and the VINGO study was approved by the Norwegian Agency for Shared Services in Education and Research (Sikt ref: 790618) and REK (2022/502016/REK Midt).

### 2.5. Consent to Participate

Written informed consent was gathered prior to enrolment in the follow-up study, including consent to merge data from the national registries mentioned above.

## 3. Results

### 3.1. Recruitment

**Figure 2:**
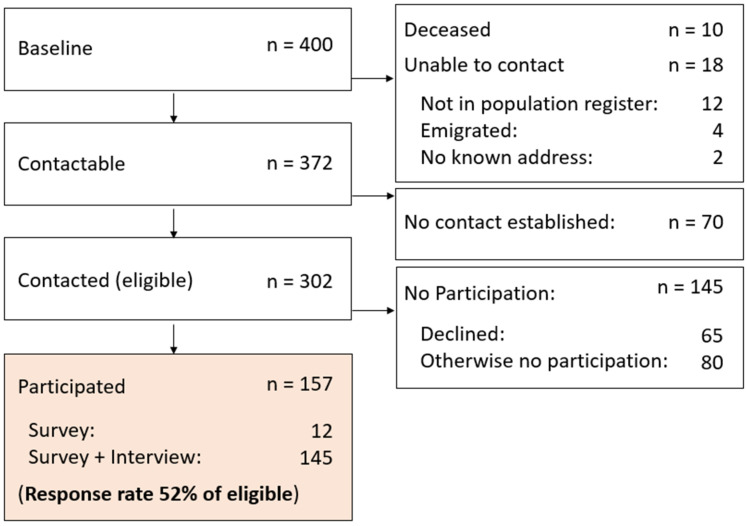
Flowchart of the VINGO study recruitment.

Contact was established with 302 (75.5%) out of the 400 participants who had consented to be contacted for future research at baseline. Of the 98 potential participants where no contact was established, 10 were deceased and 18 were without any contact information. No contact could be established with the remaining 70, despite repeated attempts. Of those contacted, 145 did not participate, either because they refused (n = 65) or never completed the survey and consent form, despite reminders (n = 80). Overall, at T2, 157 participants completed the survey, of which 145 completed the telephone interview. In total, we achieved a 52% response rate of eligible participants (where contact was established), corresponding to 39% of the original sample. For details of recruitment in the follow-up study, see Figure 2.

Sample characteristics are shown in Table 3.

### 3.2. Attrition

To assess the representativeness of the sample, we compared participants with non-participants at T2 based on key data from T1. The results of the attrition analysis are shown in Table 4.

There was a higher proportion of women in the T2 sample (67.9%) compared to T1 (57.5%). There was also a higher proportion of participants reporting Norwegian nationality. Furthermore, participants had a higher mean number of mental disorders at T2 than at T1. More specifically, they had a higher prevalence of depression, anxiety, and conduct disorder and a lower prevalence of ADHD. They also had a higher proportion of reported suicide attempts and problematic substance use. The men in the sample had a higher prevalence of experienced maltreatment.

## 4. Discussion

The VINGO study builds on a previous national cross-sectional study among adolescents in Norwegian RYC. This two-wave longitudinal study holds the potential to provide new and comprehensive knowledge on the continuity and discontinuity of health, psychosocial functioning, and quality of life within a vulnerable population [8,69]. Moreover, it will provide important knowledge regarding the transition to adulthood and the service-related factors that facilitate positive life outcomes. This knowledge is essential for stakeholders for the development of future policies and services.

The experience gained through the recruitment of participants for this follow-up study demonstrates that this vulnerable population is hard to reach. Despite extensive and systematic efforts, 52% of potentially eligible young adults from the baseline study participated in this follow-up study. There are several possible contributing factors to these challenges. Some resemble those faced by all follow-up studies, including those in the general population. For instance, baseline data may have been inaccurate in the registration of personal identification numbers, thereby leading to errors in establishing contact information at follow-up. Also, we depended on a public registry that may lack updated information regarding mobile telephone numbers. Further, emerging adulthood is a developmental phase with instability in several areas, such as habitation. This can make it difficult to reach potential participants, regardless of background characteristics.

In addition, characteristics that are more common in this population defined by prior experience with RYC compared to the general population of young adults may further explain the relatively low response rate. Results from the baseline study show a high prevalence of mental disorders. Based on knowledge of the continuity of mental health problems in the transition to adulthood [19], the prevalence of mental health problems is assumed to continue to be high. In addition, these young adults have an increased risk of living in difficult and unstable life situations related to family life, economic conditions, unemployment, and physical health [4,9,12]. These are all factors that might reduce the likelihood of responding to invitations to participate in research.

At baseline, more than half (56%) reported either daily alcohol use or that they had tried cannabis or hard drugs [24]. In the general population, weekly alcohol intake has been reported in 19% of Norwegian adolescents [70]; however, given the cultural norm of heavy episodic drinking in adolescence [71], daily alcohol consumption is likely to be less in young adulthood. Ever having tried cannabis or other illicit drugs among Norwegian adolescents was reported by 3.8% of 13–19-year-olds [72] and 14% of 17–19-year-olds [70]. Thus, alcohol and drug use among youths in RYC at baseline was very high compared to their peers. It is possible that the continuous high use of alcohol or illegal substances might have negatively influenced the motivation and ability to participate in the study for some of the potential participants.

The attrition analysis revealed differences between VINGO participants and non-participants, where participants had a higher baseline load of mental disorders and substance use and more suicide attempts and maltreatment experiences in adolescence. Thus, they have a higher risk of later physical and mental health problems. This was an unexpected finding as one would normally expect a higher response rate among more healthy individuals. It is possible that these results (except substance abuse) were caused by the higher representation of women at follow-up. The distribution of mental disorders and traumatic experiences at T1 among the participants in the current study corresponded to the reported distribution between girls and boys at T1 [1]. At T1, there were sex differences for several disorders. Compared to girls, boys had a greater risk of conduct disorder (odds ratio (OR) = 2.9), while they had a lower risk of major depressive disorder (OR = 0.3), generalized anxiety disorder (OR = 0.5), and social phobia (OR = 0.4) [1]. Another possible explanation could be that participants with a high load of traumatic experiences and mental disorders at T1 could have been motivated to participate due to a belief in receiving some kind of help through the study. It is also possible that participants might have had high motivation to participate at T2 to share their experiences to make a positive contribution to the next generation of adolescents in the care of CWSs. The results of the VINGO study have the potential to enhance our understanding about a vulnerable population and the factors that contribute to a successful transition from RYC to adulthood. The relatively low response rate, sex difference among participants, and high load of burden might pose questions about the representativeness of this study. This will need to be considered in interpreting findings from VINGO. However, this also indicates that our study has managed to reach a portion of the population that we would expect to be especially hard to reach. This might be valuable, regardless of questions about the representativeness of the sample.

At follow-up, 4 of the 170 males and 6 of the 230 females were deceased. In the general Norwegian population, among 400 persons with the same distribution of sex and age in the same period, the expected number of deaths would be 0.79 males and 0.45 females, based on mortality data from Statistics Norway. Hence, the mortality ratio compared to the general population is 4/0.79 = 5.0 for males and 6/0.45 = 13.4 for females. However, the high mortality of baseline participants is in line with findings from a Danish cohort study of 18–39-year-olds showing an adjusted hazard ratio for all-cause mortality of 3.4 (males) and 4.7 (females) for those placed in out-of-home care before age 18, corresponding to 21 and 10 extra deaths per 10,000 person-years for males and females, respectively [16]. Those with a history of out-of-home care were more likely to die from suicide, accidents (including poisoning by narcotics or hallucinogens), and cancer compared with their peers. Murray et al. reported, based on a registry study in England and Wales, increased mortality of unnatural causes among individuals with childhood out-of-home care [15].

We did not have access to data on the causes of death of the deceased in the current study. However, at baseline, 44.3% of girls and 23.2% of boys reported that they had attempted suicide [30]. Previous suicide attempts are among the strongest predictors of later suicide [73]. It is therefore possible that this could explain at least some of the excess deaths in this population. While a recent meta-study found a trend where later placement into state care increased mortality relative to those placed in state care in early childhood [74], the Danish study did not find associations between placement characteristics and mortality [16]. These studies provide us with a background for interpreting our finding of 10 out of 400 deceased at follow-up, which is an important outcome that warrants our attention. One might speculate that this death rate indicates that the follow-up by mental health and social services after the baseline study may have been insufficient to counter the effects of adverse experiences before and after placement in RYC.

It is likely that our follow-up study will conclude that many young adults with a history of living in RYC have difficulties in several life areas. Such factors may be discussed in the media and perceived as further stigmatization of an already vulnerable group. The researchers will therefore be particularly careful in the dissemination of the results to avoid unnecessary stigma and potential burden for those involved. However, it is our view that in the long run, the results from the current study may improve services and cooperation between services offered to children and adolescents living in RYC, as well as young adults with a history of living in RYC. This, in turn, should improve their health, social functioning, and QoL. Therefore, on balance, we regard it as more ethically problematic to refrain from completing the study than to actualize it.

### 4.1. Strength and Limitations

The VINGO study represents a unique opportunity to focus on a vulnerable population of emerging adults with a history of living in RYC settings. Given the scarcity of research conducted on this specific population, this study fills a critical gap in the existing literature as it provides a comprehensive perspective covering broad aspects of their lives in emerging adulthood. A notable strength of this study is the use of professionals with clinical psychiatric experience to conduct the interview portion of this study, including semi-structured diagnostic interviews. This enhances the validity of the diagnostic conclusions of the study. In addition, to capture a comprehensive understanding of the respondents’ perceptions, subjective experiences of inter-service cooperation were obtained. This mixed-method design using both quantitative measures and qualitative interviews with a selected subset of participants should enhance our understanding of this population at a critical transition in life. This methodology has the potential to provide rich, nuanced insights into participants’ experiences, which will provide great value for stakeholders and service providers including CWSs and health workers.

The relatively low response rate is a limitation of this study, especially the low rate of male participants compared to females. However, the attrition analysis shows that we managed to include individuals who had a high load of mental health problems at baseline. It could be argued that diagnostic assessment would have been improved further by conducting in-person interviews instead of by telephone. However, conducting in-person interviews all over the country would have been very expensive, so this was impossible to achieve given the funding of our study. Further, this would probably have diminished the response rate further. Indeed, research has reported telephone interviews to be of good quality compared to face-to-face interviews [75,76].

### 4.2. Theoretical Implications

The challenging recruitment of participants to the VINGO study suggests an alternative understanding of how emerging adulthood affects individuals with a history of RYC. In the recruitment process, we observed that most of the eligible young adults had severe psychosocial struggles and difficulties in adhering to appointments to complete participation in this study. Furthermore, the mortality rate was high. The developmental stage of emerging adulthood is expected to represent increasing independence and maturity of the individual. However, our findings could imply that young adults with a history of RYC follow other trajectories into adulthood. The life course framework for studying vulnerability could be an alternative theoretical approach [77]. This perspective emphasizes vulnerability as a dynamic process of stress and resources, leading to various trajectories. Young people aging out of RYC have a high burden of adversities, and the developmental stage of emerging adulthood could represent a significant additional stressor promoting difficulties in several life areas.

### 4.3. Implications for Research

Research on hard-to-reach populations is challenging. Such research demands additional resources regarding both time to complete data collection and funding to ensure the necessary follow-up of potential participants. However, the fact that the individuals are hard to reach means that there is a lack of an evidence base to build upon when planning and implementing measures to improve their lives. We recommend extensive and flexible recruitment strategies for research. Examples that could be important for success are (1) personal contact; (2) possibility of audio guidance to complete surveys; (3) reminders by SMS, emails, or phone calls; and (4) the use of social media to reach participants.

### 4.4. Implications for Services

The eligible participants of the VINGO study were found to have poor personal economy, low educational levels, high rates of unemployment, and disability benefits. In addition to the high risk of poor health, this suggests that they need a range of services. Our experience of challenging recruitment indicates that they need more person-centered services, such as mentors in daily life, frequent reminders of appointments, and personal guidance with regard to reaching service providers.

## 5. Conclusions

Recruitment of hard-to-reach populations for research is important for developing tailored services. Our results from the VINGO study show that we managed to reach participants with high burden loads. The data collected give a unique opportunity to study a vulnerable population of emerging adults. Given the scarcity of research conducted on this specific population, this study fills a critical gap in the existing literature as it provides a comprehensive approach covering broad aspects of life.

## Figures and Tables

**Table 1:** Overview of objectives and selected research questions of the VINGO study.

Objectives	Selected Research Questions
Mental health	Is there a change at individual and group levels in the prevalence of mental disorders from baseline to follow-up?
	What are the prevalence and predictors of trauma- and stressor-related disorders and how are they associated with other mental and physical problems and other important life outcomes?
Physical health	How is child maltreatment at different time points between 0 and 18 years of age associated with reported headaches and physical pain among young adults with a history of living in RYC?
Child maltreatment	Does the association between childhood maltreatment and psychosocial functioning in young adulthood follow a dose-dependent relationship and/or a time- and type-sensitive pattern?
Alcohol and drug use	How is the stability and change of alcohol and illicit drug use characterized from adolescence to adulthood?
Social support	Is there an association between adolescents’ network size and specific support persons when living in RYC and perceived social support in young adulthood?
Life situation	What are the predictors of unemployment and qualifying for disability benefits among young adults with a history of living in RYC?
Service use	Do individual factors (e.g., gender, mental health at baseline, exposure to child maltreatment) predict the type and duration of received aftercare after leaving RYC?
	Do type and duration of aftercare predict successful transition to adulthood (completion of education, social function, employment, income)?
Experiences with services	Can user-reported experiences with health and social care services predict current mental health status?

**Table 2:** Domains and measures.

Domain	Measure	Items	S	I	Used in HUNT/ CAP Study	Satisfactory Reliability and Validity Documented
Sociodemographic information	Gender, age, education, income, employment	22	X		X	Not applicable
Family and social relations, employment, and education	Items from the adult version of the Achenbach System of Empirically Based Assessment (ASEBA) [41]	45	X			Yes, in international samples [41]
Timeline	Living arrangements and number of residential moves since baseline interview	1–15		X		Not applicable
Mental disorders	M.I.N.I. INTERNATIONAL NEUROPSYCHIATRIC INTERVIEW (MINI plus) [42] for current and lifetime mental disorders	45		X		Yes, in international [42] and Norwegian samples [43]
PTEs/PTSD	TRauma And Ptsd Screening (TRAPS) questionnaire, and PTSD Checklist for DSM-5 (PCL-5) [44,45]	35		X		Yes, in international samples [45]
CPTSD	Interpersonal Trauma Questionnaire (ITQ) [46]	9		X		Yes, in international samples [46,47]
Dissociation	Shutdown Dissociation scale (Shut-D) [48]	13	X			Yes, in international samples [48]
Personality traits	DSM and ICD Personality Questionnaire (DIP-Q) [49,50,51]; in the current study, the 102 items referring to DSM-IV personality disorders were included	102		X		Yes, in Scandinavian samples [50,51,52]
Physical health	Somatic disorders, pain, sleep, physical activity, and medication use	47–113	X		X	Validity by use in the Norwegian HUNT study
Impact of the COVID-19 pandemic	Items addressing how the COVID-19 pandemic influenced work or education, mental health, and feelings of loneliness.	3	X			No, custom-made for the present study
Quality of life	World Health Organization Quality of Life Questionnaire (WHOQoL-BREF) assessing physical, psychological, social relationship, and environment domains of QoL [53]	26	X			Yes, in international [54,55] and Norwegian samples [56,57]
Exposure to child maltreatment	Maltreatment and Abuse Chronology of Exposure (MACE) scale assessing ten domains of child maltreatment [58,59]	55	X			Yes, in international [58] and Norwegian samples [59]
Social support	Social Support Questionnaire (SSQ) (short form) [60]	5	X			Yes, in Norwegian samples [60]
Self-esteem	The revised version of the Self-Perception Profile for Adolescents (SPPA) [61,62] assessing five subdomains of self-esteem and global self-worth	30	X			Yes, in Norwegian samples [60]
Psychosocial functioning	GAF-score 0–100, scored by the interviewer based on total interview	1		X		Yes, in international [63] and Norwegian samples [64]
Service use and experiences	Items from the HUNT/CAP study [65,66] on experiences with general practitioners and specialized healthcare services	26	X		X	Validity by use in the Norwegian HUNT study
	Child and Adolescent Services Assessment (CASA) addressing the use of aftercare, primary and specialist health services, CWSs, and social services [67,68], accessible in a Norwegian context; it comprises current and lifetime service use, experiences with services in the last 6 months, and barriers to service use	Part 1:22 Part 2: 24 × 0–3 Part 3: 43		X		Yes, in international samples [68]
	Custom-made questions concerning experience with cooperation between services, the subjective need for alternative schooling, and any services participants felt they should have been offered but were not	33 (q) 5 (I)	X	X		No, custom-made for the present study
	Qualitative interview (see text)					Not applicable

S: Survey; I: Interview; HUNT study: The Trøndelag Health Study—a longitudinal study of the general population in the county Trøndelag in Norway; CAP study: The Child and Adolescent Psychiatry study—a longitudinal study of individuals with a history of psychiatric problems as adolescents; PTEs: Potentially traumatic events; PTSD: Post-traumatic stress disorder; CPTSD: Complex PTSD.

**Table 3:** Sample characteristics.

	n	%
Female sex	107	68.2
Age (mean)	25.4 (range: 22–30 y)	
Birth country Norway	145	92.4
Marital status		
Married/living with partner	60	38.2
Widowed	2	1.3
Separated/divorced	5	3.1
Never been married/lived with partner	48	30.6
Children	45	29.2
Education completed		
1–10 years	62	39.5
11–12 years	37	23.6
13 years	23	14.6
Certificate of apprenticeship	22	14.0
1–3 years of higher education	6	3.8
≥4 years of higher education	4	2.5
Dropped out of education	111	70.7
Personal economy		
Bad/very bad	70	44.6
Okay	56	35.7
Good/very good	31	19.7
Employed	63	40.1
In education	23	14.6
Military service	2	1.3
Other types of occupation	10	6.4
Disability benefits (100%)	34	21.7

**Table 4:** Attrition.

T1 Data	Participants T2 (n = 157)		Non-Participants T2 (n = 243)	
Age at T1, mean (SD)	16.9 (1.2)		16.7 (1.4)	
Female sex, n (%)	107 (68.2)		123 (50.8)	
Country of origin, Norway, n (%)	135 (86.5)		171 (70.7)	
DSM IV diagnoses, mean (SD)	2.6 (2.6)		2.0 (2.3)	
	Men	Women	Men	Women
Any depressive disorder, n (%)	9 (20.0)	51 (54.3)	22 (23.7)	43 (42.2)
Any anxiety disorder, n (%)	12 (26.7)	42 (44.7)	26 (28.0)	37 (36.3)
ADHD, n (%)	19 (38.0)	26 (24.3)	44 (37.0)	40 (32.8)
Conduct disorder, n (%)	14 (31.1)	15 (16.0)	25 (26.9)	9 (8.8)
Self-reported suicide attempt, n (%)	17 (38.6)	43 (46.7)	15 (16.1)	43 (42.2)
Any maltreatment ^1^, n (%)	29 (64.4)	73 (77.7)	54 (58.1)	80 (78.4)
Problematic substance use ^2^, n (%)	23 (59.0)	53 (59.6)	44 (54.3)	51 (54.8)
Physical well-being, mean ^3^, n (SD)	69.9 (19.4)	43.4 (23.4)	69.7 (20.9)	55.4 (24.9)
Emotional well-being, mean ^3^, n (SD)	71.5 (19.1)	58.3 (24.9)	71.5 (22.2)	60.9 (24.4)
Self-esteem, mean ^3^, n (SD)	55.0 (26.0)	40.9 (24.7)	59.9 (27.4)	43.4 (24.2)
Friends, mean ^3^, n (SD)	67.3 (23.7)	65.3 (23.8)	71.6 (20.1)	68.3 (21.2)

^1^: experienced sexual abuse or violence or witnessed violence, ^2^: daily use of alcohol or use of cannabis or hard drugs, ^3^: KINDL-r quality of life scale 0–100.

## Data Availability

The data presented in this study are available upon reasonable request from the corresponding author. The data are not publicly available due to ethical restrictions.
